# Improved PrEP Awareness and Use among Trans Women in San Francisco, California

**DOI:** 10.1007/s10461-021-03417-3

**Published:** 2021-08-14

**Authors:** Erin C. Wilson, Christopher J. Hernandez, Susan Scheer, Dillon Trujillo, Sean Arayasirikul, Sofia Sicro, Willi McFarland

**Affiliations:** 1 Center for Public Health Research, San Francisco Department of Public Health, 25 Van Ness Avenue, San Francisco, CA 94102 USA; 2 HIV Epidemiology Section, San Francisco Department of Public Health, San Francisco, CA USA; 3 Department of Pediatrics, University of California San Francisco, San Francisco, CA USA; 4 Department of Psychiatry, University of California San Francisco, San Francisco, CA USA; 5 Department of Biostatistics and Epidemiology, University of California San Francisco, San Francisco, CA USA

**Keywords:** Transgender women, Pre-exposure prophylaxis, PrEP, HIV, PrEP cascade

## Abstract

Transgender women face a serious risk of HIV infection. Despite this, there is limited knowledge and use of Pre-exposure prophylaxis (PrEP). We measured the continuity of prevention across services in the PrEP cascade and correlates of PrEP use among trans women in San Francisco enrolled in the 2019/20 National HIV Behavioral Surveillance Study. Knowledge and use of PrEP among trans women in San Francisco increased in recent years; almost all (94.0%) had heard about PrEP, 64.7% had discussed PrEP with a healthcare provider, and 44.8% had taken PrEP in the past 12 months. PrEP use was associated with participation in a PrEP demonstration project (aOR = 31.44, p = 0.001) and condomless receptive anal intercourse (aOR = 3.63, p = 0.024). Injection drug use was negatively associated (aOR = 0.19, p = 0.014). Efforts are needed to combat the gender-based stigma and discrimination faced by trans women, which can result in avoidance and mistrust of the medical system.

## Resumen

Las mujeres trans enfrentan un grave riesgo de infección por el VIH. A pesar de ello, hay conocimiento y utilización limitada de la profilaxis previa a la exposición (PrEP). Medimos la continuidad de prevención a través de los servicios en la cascada de PrEP y los correlatos del uso de PrEP entre mujeres trans en San Francisco inscritas en el Estudio Nacional de Vigilancia del Comportamiento del VIH en 2019/20. El conocimiento y el uso de PrEP entre las mujeres trans en San Francisco aumentó en los últimos años; casi todas (94.0%) habían escuchado sobre PrEP, el 64.7% habían hablado de PrEP con un proveedor de atención médica y el 44.8% había tomado PrEP en los últimos 12 meses. El uso de PrEP se asoció con el uso actual con participación en un proyecto de demostración de PrEP (ORa = 31.44, p = 0.001) y el coito anal receptivo sin condón (ORa = 3.63, p = 0.024). El uso de drogas inyectables se asoció negativamente (ORa = 0.19, p = 0.014). Se necesitan esfuerzos para combatir el estigma y la discriminación basados en el género que enfrentan las mujeres trans, que pueden resultar en la evitación y desconfianza en el sistema medico.”

## Introduction

Transgender women (henceforth abbreviated as trans women) face a substantial and continued risk for HIV infection that has been well documented around the world [1–4]. This is particularly concerning during a period in the United States when national efforts to reduce incident HIV infections and “End the Epidemic” have been intensified and successful in many areas. For example, in San Francisco, a city that has embarked on an aggressive and increasingly successful HIV prevention campaign to ‘Get to Zero’ [5, 6], new HIV diagnoses have fallen dramatically in recent years [7]. However, the same success has not been seen among trans women. Between 2014 and 2019, new HIV diagnoses declined by 31% in the city overall and across most demographic groups. Yet in 2019, trans women accounted for 7% of new diagnoses, up from a range of 3–4% between 2014 and 2018 and well above their relative numbers in the population as a whole [8–10]. Additionally, HIV prevalence is particularly high among trans women worldwide [1, 11]. In San Francisco, HIV prevalence is estimated to be between 32 and 41% among trans women [4, 12], higher than any other population.

Prior studies find that trans women, compared to other populations, have reported limited knowledge of and uptake of Pre-exposure prophylaxis (PrEP), a widely recognized and proven cost-effective strategy to reduce HIV acquisition [13–15]. For example, in 2013, only 13.7% of trans women in San Francisco reported they had heard of PrEP and only one of those was willing to use PrEP for HIV prevention [16].

Many of the barriers to PrEP use faced by trans women are unique to this population including discrimination and stigma based on transgender identity [14]. Past experiences of stigma and discrimination specific to gender identity (e.g., experiences being mis-gendered and being faced with transphobia in the medical system and by providers) make trans women unwilling to seek health care in general [14, 17], and specifically regarding PrEP, concerns about interactions with hormone use and/or the desire to prioritize hormone therapy over HIV prevention and treatment therapies [18, 19] impact decisions regarding PrEP use. Recent data from San Francisco found that trans women were less likely than men who have sex with men (MSM) to have engaged in each step along the PrEP cascade; 79% of trans women reported having heard of PrEP compared to 97% of MSM and 15% reported using PrEP compared to 40% of MSM [20].

These barriers to PrEP are exacerbated for Black trans women and are of particular concern given that Black trans women are disproportionately affected by HIV [21]. A recent meta-analysis found that Black trans women compared to white trans women and trans women of all other races had a significantly higher HIV prevalence estimate [22] and the National HIV Surveillance System reported that the majority (51%) of all new HIV diagnoses among trans women in the United States are among Black trans women [23]. The risk of HIV infection for Black trans women and other trans women of color is highly affected by the intersection of discrimination and stigma they face due to sexism, racism, homophobia and transphobia [11] This intersectionality of discrimination and oppression results in both high HIV prevalence as well as barriers to and trust in HIV prevention methods such as PrEP [24, 25].

In 2019, for the first time, trans women were included in the Centers for Disease Control and Prevention National HIV Behavioral Surveillance Study (NHBS) in San Francisco. NHBS has cyclically surveyed MSM, people who inject drugs, and high risk heterosexuals since 2003. We conducted a secondary analysis of the first NHBS wave for trans women using data from the PrEP-eligible participants to ascertain engagement in the PrEP cascade and to examine if engagement has improved among trans women in recent years. Additionally, we examined risks for HIV and correlates of PrEP to identify barriers to optimal PrEP access and adherence and to identify potential intervention strategies.

## Methods

This is a secondary analysis of data from the first cycle of the National HIV Behavioral Surveillance (NHBS) conducted for trans women in San Francisco from July 2019 to February 2020. For this analysis on PrEP engagement, participants known to have HIV were excluded as they are no longer PrEP-eligible.

Respondent-driven sampling (RDS) was used to obtain a diverse, community-based sample of trans women. Fifteen trans women, diverse with respect to demographic characteristics, were enlisted to recruit their peers. These initial 15 “seeds” were instructed to refer to other eligible trans women, namely 18 years of age or older, resident of San Francisco, and identifying as a trans woman (i.e., as a woman and gender other than male as assigned at birth). Eligible recruits completed an interviewer-administered questionnaire, provided blood specimens for HCV and HIV antibody and HIV viral load testing, and in turn, were asked to refer up to 10 other trans women to the study. Participants received $100 for completing the study activities and an additional $25 for each eligible peer referral enrolled into the study. Recruitment continued until the sample size (N = 201) was met and the composition of the sample stabilized with respect to demographic characteristics.

### Measurements

In addition to being asked if they were previously diagnosed with HIV, participants were tested with the Oraquick® HIV Rapid Antibody Test (OraSure Technologies, Bethlehem, PA) to detect HIV antibodies.

An interviewer-administered survey included questions on demographic characteristics, substance use, risk behaviors, and indicators of PrEP engagement. An interviewer-administered survey included questions on demographic characteristics, substance use, risk behaviors, and indicators of PrEP engagement. Participants were asked to report use of any drugs that were not prescribed to them in the past 12 months (dichotomized as using or not using marijuana, methamphetamine, crack cocaine, powder cocaine, downers, painkillers, heroin, poppers, or something else and injected or not injected). Survival or exchange sex was assessed by asking participants if they had received money or drugs in exchange for sex in the past 12 months. Sexual behavior was ascertained by asking if participants had had receptive anal, insertive anal, and vaginal sex in the past 12 months as well as asking participants about each of these behaviors with their three last sexual partners. For each of these partners, participants were asked “What was your partner’s HIV status?” and whether or not a condom was used for each sexual behavior.

Hormone use was categorized as “yes” for those who reported currently being on any hormones, which could have included non-prescribed hormones. Participants were also asked if they had enrolled in a demonstration project implemented during the study period in San Francisco to improve PrEP uptake and adherence specifically with trans populations. PrEP engagement (the PrEP cascade) included awareness of PrEP, discussing PrEP use with a medical provider, PrEP use, and adherence and persistence in using PrEP. To measure awareness, participants were asked “Before today, have you ever heard of PrEP?” Discussion with a provider was ascertained by asking “In the past 12 months, have you had a discussion with a health care provider about taking PrEP?” PrEP use was determined from the question “In the past 12 months, have you taken PrEP to reduce the risk of getting HIV?” To determine adherence, participants were asked “When you took PrEP in the past 12 months, did you take it every day, almost every day, or less often?” Participants who answered every day or almost every day were classified as highly adherent. Finally, to determine persistence, participants were asked “Did you take PrEP for at least 2 months in a row?”.

### Statistical Analysis

Descriptive statistics (i.e., frequencies and proportions) were used to summarize demographic characteristics (e.g., age, race/ethnicity, education) and PrEP engagement indicators. Bivariate logistic regression models were used to find correlations between participant characteristics and each step along the PrEP cascade, using p < 0.05 as the level for significance. Condomless intercourse (insertive or receptive vaginal or anal) was collapsed so that responses of “No” and “Not applicable” were in the same category as they both are risk-reduction practices. A multivariable model was built with correlations that were associated with PrEP use at the bivariate p < 0.05 level. Observations that were co-linear were removed and the observation with a lower p-value in the model were retained. In addition, we controlled for age, race/ethnicity, and education.

### Ethical Considerations

Participants who tested positive for HIV, HCV, or other STIs were provided counseling and resources for linkage to appropriate care and treatment. The study protocol was reviewed and approved by the Institutional Review Board (IRB) at the University of California, San Francisco (UCSF) (#17-24062). All participants provided written informed consent.

## Results

### Participant Characteristics

Overall, 201 trans women participated in the study. Participants who were living with HIV were excluded (n = 85) and the remaining 116 participants without HIV infection, and therefore PrEP-eligible, were included in this secondary analysis. Of these, the mean age was 47.7 years (median 43, interquartile range [IQR] 32–52) (Table 1). Participants were diverse with respect to race/ethnicity; 42.2% identified as Hispanic/Latinx, 23.3% identified as other, 22.4% identified as White, and 12.1% identified as Black/African American. For gender, the majority (68.3%) identified as a trans woman, while 13.5% identified as woman, and 18.3% had another identity. In terms of education, 49.1% had a high school education/GED or less, 17.2% had some college or a technical degree, and 33.6% completed a college degree or more. Most participants (56.0%) were living at or below the San Francisco poverty level, 73.3% were unemployed at the time of this survey, 70.7% were unstably housed or homeless.

**Table 1:** Characteristics of HIV-negative trans women, National HIV Behavioral Surveillance (NHBS), San Francisco, CA, 2019 (N = 116)

Characteristic	N	%
Age (years)	–	–
Median (IQR): 43 (32–52)	–	–
Race/ethnicity		
White	26	22.4
Black or African American	14	12.1
Hispanic/Latino/a	49	42.2
Other	27	23.3
Gender identity		
Trans woman	71	68.3
Woman	14	13.5
Other identity	19	18.3
Education		
High school, GED, or less	57	49.1
College degree and above	39	33.6
Some college/technical degree	20	17.2
Annual income	51	44.0
Above poverty limit	65	56.0
At or below poverty limit		
Employed		
No	85	73.3
Yes	31	26.7
Unstably housed, homeless		
No	34	29.3
Yes	82	70.7
Ever incarcerated		
No	48	41.4
Yes	68	58.4
Sexual partner with HIV		
No	112	96.6
Yes	4	3.4
Injection drug use, last 12 months		
No	106	91.4
Yes	10	8.6
Number of sexual partners, last year		
Mean: 18.8	–	–
Median (IQR): 4 (1–7)	–	–
Condomless insertive anal sex, last year		
No	92	79.3
Yes	24	20.7
Condomless receptive anal sex, last year		
No	55	47.4
Yes	61	52.6
Condomless receptive vaginal sex, last year		
No	109	94.0
Yes	7	6.0
Exchange sex, last year		
No	71	61.2
Yes	45	38.8
Number of exchange partners		
Mean 38.8		
Median (IQR): 5 (2–30)		
Sexual partner using PrEP		
No	106	91.4
Yes	10	8.6
Participated in PrEP demonstration project for trans people in SF		
No	79	68.1
Yes	37	31.9

### HIV-Related Risk Factors

Only 3.4% of participants had a sexual partner living with HIV (Table 1). Participants reported an average 18.8 sexual partners (median 4, IQR 1–7). Additionally, 38.8% of participants engaged in exchange sex in the last year. Over half of participants (52.6%) engaged in condomless receptive anal sex in the past year, while 20.7% engaged in condomless insertive anal sex, and 6.0% engaged in condomless receptive vaginal sex. Ten participants (8.6%) reported injection drug use in the last 12 months.

### PrEP Indicators

**Fig. 1:**
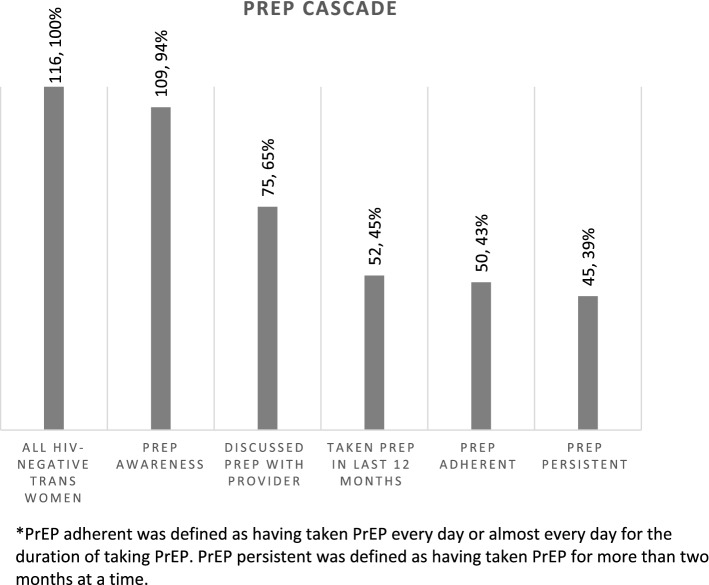
Engagement of trans women with PrEP, San Francisco, 2019 (n = 116). *PrEP adherent was defined as having taken PrEP every day or almost every day for the duration of taking PrEP. PrEP persistent was defined as having taken PrEP for more than two months at a time

Almost all (94.0%) participants had heard of PrEP (Fig. 1). Seventy-five (64.7%) reported discussing PrEP with a healthcare provider during a healthcare appointment. Fifty-two (44.8%) participants reported taking PrEP in the last 12 months and 45 (38.8%) reported taking it for more than two months at a time. Fifty (43.1%) of those who had taken PrEP reported taking it every single day or nearly every single day, indicating a high adherence to PrEP.

### Demographic and Behavioral Correlates of PrEP Indicators

Increased awareness of PrEP was associated with seeing PrEP advertisements (OR 4.64, 95% CI 1.26–17.16, p = 0.021) and hormone use (OR 7.98, 95% CI 1.46–43.59, p = 0.017) (Table 2). Lower odds were found for those seropositive for HCV antibodies (OR 0.19, 95% CI 0.039–0.97, p = 0.046). Discussing PrEP with a healthcare provider was significantly associated with receiving healthcare in a clinic that offers PrEP (OR 10.5, 95% CI 2.97–37.04, p = 0.001) and participation in the STAY study (OR = 10.5, 95% CI: 2.97–37.04, p = 0.001), a PrEP demonstration project implemented in San Francisco during the time of this study. Having taken PrEP in the last twelve months was significantly associated with having at least one sexual partner who was on PrEP (OR 6.00, 95% CI 1.15–31.2, p = 0.033), being currently insured (OR 3.86, 95% CI 1.50–9.90, p = 0.005), and engaged in condomless receptive anal sex in the last twelve months (OR 2.23, 95% CI 1.05–4.73, p = 0.036). PrEP use was also associated with having participated in the PrEP demonstration project (OR = 26.05, 95% CI: 8.17–83.01, p = 0.001). Lower odds of PrEP use in the last 12 months was associated with injection drug use (OR 0.25 95% CI 0.09–0.73, p = 0.011) and being HCV seropositive (OR 0.22 95% CI 0.059–0.81, p = 0.023). Correlates with adherence were not found due to the small number of observations.

**Table 2:** Associations with PrEP indicators, trans women, National HIV Behavioral Surveillance (NHBS), San Francisco, CA, 2019 (N = 116)

Predictor variables	Aware of PrEP OR (95% CI), p-value	Discussed PrEP with provider OR (95% CI), p-value	Took PrEP in last 12 months OR (95% CI), p-value	Took PrEP in last 12 months Adjusted OR (95% CI)
Age	0.93 (0.87–0.99), 0.038^1^	0.96 (0.93–0.99), 0.020	0.96 (0.94–0.99), 0.025	0.97 (0.92–1.01), 0.167
Race/ethnicity				
White	Reference	Reference	Reference	Reference
Black/African American	0.52 (0.03–9.00), 0.653	0.40 (0.09–1.62), 0.199	0.65 (0.17–2.47), 0.525	0.56 (0.07–4.42), 0.583
Hispanic/Latinx	0.45 (0.48–4.25), 0.486	0.44 (0.15–1.28), 0.129	0.80 (0.31–2.10), 0.657	0.76 (0.17–3.44), 0.721
Mixed/other	1.04 (0.06–17.55), 0.978	6.00 (0.18–2.02), 0.409	1.46 (0.49–4.30), 0.494	0.97 (0.18–5.11), 0.972
Education				
Up to high school	Reference	Reference	Reference	Reference
College level	0.66 (0.13–3.49), 0.631	1.00 (0.42–2.37), 0.99	0.86 (0.38–1.95), 0.715	0.36 (0.10–1.31), 0.121
Bachelors or higher	1.05 (0.10–10.77), 0.964	0.61 (0.21–1.72), 0.353	0.74 (2.63–2.08), 0.570	0.47 (0.10–2.13), 0.325
Saw PrEP advertisements	4.64 (1.26–17.16), 0.021	1.19 (0.855–1.67), 0.295	1.38 (0.99–1.92), 0.051	–
Used post-exposure prophylaxis	1.35 (0.15–11.83), 0.787	1.95 (0.65–5.78), 0.227	1.83 (0.71–4.77), 0.214	–
At least one sexual partner who was on PrEP	Omitted^2^	Omitted	6.00 (1.15–31.2), 0.033	–
Currently on hormones	7.98 (1.46–43.59), 0.017	3.73 (1.58–8.81), 0.003	3.86 (1.50–9.91), 0.005	1.89 (0.53–6.75), 0.329
Currently has health insurance	1.34 (0.15–12.16), 0.791	2.37 (0.74–7.59), 0.147	3.86 (1.50–9.90), 0.005	0.83 (0.14–4.71), 0.829
Participated in PrEP demonstration project for trans people in SF	Omitted^2^	10.5 (2.97–37.04), 0.001	26.05 (8.17–83.01), 0.001	31.44 (7.86–125.78), 0.001
Received healthcare in last year at a clinic where PrEP is offered	1.13 (0.21–6.15), 0.889	10.5 (2.97–37.04), 0.001	1.34 (0.41–4.38), 0.625	–
Injection drug use, last 12 months	0.31 (0.066–1.53), 0.153	0.88 (0.35–2.25), 0.804	0.25 (0.09–0.73), 0.011	0.15 (0.03–0.82), 0.029
HCV seropositive	0.19 (0.039–0.97), 0.046	1.00 (0.34–2.94), 0.996	0.22 (0.059–0.81), 0.023^3^	–
Arrested or held	0.1 (0.0098–1.05), 0.055	1.36 (0.63–2.94), 0.423	0.7 (0.33–1.47), 0.347	–
Condomless receptive intercourse	Omitted^2^	1.01 (0.31–3.22), 0.984	1.93 (0.67–5.56), 0.225	–
Condomless receptive anal sex	0.82 (0.17–3.84), 0.804	2.00 (0.93–4.33), 0.078	2.23 (1.05–4.73), 0.036	3.63 (1.18–11.13), 0.024
Condomless receptive vaginal sex	Omitted^2^	0.71 (0.15–3.36), 0.669	0.92 (0.19–4.3), 0.914	–
Condomless insertive anal sex	0.632 (0.11–3.49), 0.598	0.71 (0.15–3.36), 0.669	1.30 (0.52–3.19), 0.568	–
Condomless insertive vaginal sex	0.54 (0.058–4.99), 0.587	1.30 (0.32–5.33), 0.712	1.96 (0.52–7.34), 0.320	–

^1^Highlighted values were significant at the p $\le$ 0.05 level

^2^Omitted due to small sample size or perfect match between outcome and predictor variables

^3^HCV seropositivity not included in final adjusted model due to collinearity with injection drug use

### Multivariable Model

OraQuick HCV antibody test was dropped from the analysis because of its collinearity with a history of injection drug use. Among characteristics that were collinear with one another, we retained the characteristic that had the lower p-value at the bivariate level in the multivariable model. Participating in the PrEP demonstration project for trans people was also removed in order to improve the goodness of fit of the model. After controlling for age, race/ethnicity, and education, PrEP use in the last 12 months was positively associated with having participated in the PrEP demonstration project (aOR = 31.44, 95% CI = 7.86–125.78, p = 0.001) and having condomless receptive anal intercourse (aOR = 3.63, 95% CI = 1.18–11.13, p = 0.024) (Table 2). Having a history of injection drug use was negatively associated with PrEP use in the last 12 months (aOR = 0.15, 95% CI = 0.03–0.82, p = 0.029).

## Discussion

Nearly all trans women participating in the 2019/20 NHBS study of trans women in San Francisco reported being aware of PrEP and nearly half had used PrEP in the last 12 months. These data show substantial increases along all indicators on the PrEP cascade compared to recent findings in previous surveys of trans women in San Francisco. For example, among trans women enrolled in another study in the San Francisco Bay Area from 2016 to 2018, 79% were aware of PrEP and only 14.7% reported using PrEP [20].

Our findings also point to sexual risk behavior as a strong facilitator of PrEP use. Trans women who engaged in condomless receptive anal sex were most likely to have been on PrEP in the last 12 months, speaking to the high self-efficacy trans women have for caring for their own sexual health. Further, being part of a PrEP demonstration project (the STAY study), which focused on making PrEP assessible to trans communities was positively associated with having taken PrEP in the last 12 months. Working to get trans women in culturally-relevant primary care services and meeting their PrEP-specific needs outside primary care, as was done in the demonstration project, can increase their use of PrEP.

Previous research has found that significant racial/ethnic disparities exist in PrEP knowledge and use [26–28] particularly among Black/African Americans and the Latinx community and that these disparities are pronounced among trans women [20]. Black trans women made up 15% of the STAY study population, and, in this study, 11% of the participants who reported they were also a STAY study participant were Black (data not shown). It is encouraging that these results and improvements along the PrEP cascade were observed across all racial groups in a diverse sample of trans women where over three-quarters were people of color.

Despite the encouraging findings regarding PrEP engagement, trans women in our analysis who injected drugs in the last year were significantly less likely to be engaged in PrEP. This finding is consistent with data from San Francisco showing very low engagement in PrEP among people who inject drugs [29]. Trans women in our analysis also reported significant socio-economic barriers and high risk for HIV. High rates of condomless sex, sex work, and injection drug use were documented. Furthermore, over half of this population were at or below the San Francisco poverty level, had ever been incarcerated, nearly three quarters were unstably housed, and educational attainment and employment were low. Each of these social determinants of health have been previously shown to be associated with HIV infection, barriers to HIV treatment, and lower use of effective prevention strategies including PrEP [7, 30–32].

Our study is subject to a number of limitations. First, RDS may not produce representative samples of trans women. While RDS is a strategy purported to reach otherwise hard-to-reach participants, the majority of our participants reported an annual income below the poverty level. This has been the case with other local surveys of trans women that have used RDS [4, 33]. RDS that relies on peer referrals that are incentivized with cash stipends can lead to recruitment from social networks of lower SES [34, 35]. As such, results from our study may not be generalizable to trans women with higher incomes in San Francisco or elsewhere. Second, programs, policies, and resources available to trans women in San Francisco, including PrEP outreach, may also limit the generalizability of our findings to other locations. San Francisco has actively promoted PrEP as part of its “Getting to Zero HIV” campaign and therefore awareness may be higher in our city compared to elsewhere. Third, this was an interviewer-administrated survey and may be subject to social desirability bias. However, this limitation may be mitigated by the use of well-trained, skilled and experienced interviewers who have an established rapport with the study population as utilized in this NHBS cycle. Finally, we were unable to stratify our sample to examine correlations within important sub-groups due to a limited sample size.

## Conclusion

Knowledge of and uptake of PrEP among trans women in San Francisco has dramatically increased in the last few years. Trans women in San Francisco may have benefited from the overall citywide push to roll out PrEP in recent years. PrEP availability and access is one of the priority goals of the SF Getting to Zero effort, and trans women, a population recognized as being previously underserved, have been prioritized for media campaigns and outreach in recent years [5, 20] and specific efforts tailored to the trans community have been implemented. Sustainability of such efforts may help maintain high engagement in PrEP, especially those that build upon trans women’s resiliency to meet their own sexual health and gender affirming care needs. Despite some encouraging findings, efforts must continue to address the barriers to PrEP access and use, especially among trans women who also inject drugs. Furthermore, efforts to address significant specific gender-based stigma and discrimination faced by trans women, which results in health care avoidance and distrust are needed. An area of further research is the engagement in PrEP of the partners of trans women who are likely at high risk for HIV given the extremely prevalence among trans women in San Francisco and other cities [36]. To reach optimal PrEP uptake and ‘get to zero’ for everyone, steps are needed to ensure that particularly vulnerable populations such as trans women and their partners are reached and their specific barriers to PrEP awareness, trust and uptake are addressed.
